# Neuropathic painful complications due to endopelvic nerve lesions after robot-assisted laparoscopic prostatectomy: Three case reports

**DOI:** 10.1097/MD.0000000000018011

**Published:** 2019-11-15

**Authors:** Marco Cascella, Giuseppe Quarto, Giovanni Grimaldi, Alessandro Izzo, Raffaele Muscariello, Luigi Castaldo, Barbara Di Caprio, Sabrina Bimonte, Paola Del Prete, Arturo Cuomo, Sisto Perdonà

**Affiliations:** a Division of Anesthesia and Pain Medicine; b Uro-Gynecological Department; c Direzione Scientifica, Istituto Nazionale Tumori, IRCCS - Fondazione G. Pascale, Naples, Italy.

**Keywords:** prostate cancer, robot-assisted laparoscopic prostatectomy, neuropathic pain, hyperalgesia, obturator nerve, pudendal nerve

## Abstract

**Rationale:**

Robot-assisted laparoscopic prostatectomy (RALP) is the most frequent strategy used for the surgical remedy of patients with localized prostate cancer. Although there is awareness about potential patient positioning nerve injuries, iatrogenic nerve lesions are less described in the literature. Here, we report 3 cases of patients who presented with neuropathic painful complications due to RALP-associated nerve lesions.

**Patient concerns:**

A 62-year-old patient (case 1), a 72-year-old male (case 2), and a 57-year-old patient (case 3) presented at the clinic with symptoms of neuropathic pain after RALP surgery.

**Diagnosis:**

Patients were diagnosed with a potential injury of different branches of the pudendal nerve (cases 1 and 2), and left obturator nerve (case 3).

**Interventions:**

Patients underwent multimodal pharmacologic treatment through pregabalin, weak opioids, strong opioid, paracetamol, and adjuvants. In cases 2 and 3, a multidisciplinary approach was needed. As the patients responded to conservative treatment, invasive approaches were not necessary.

**Outcomes:**

After treatment, the patients of case 1 showed pain relief after 4 days, paresthesia resolved in 15 days, whereas the anal crushing sensation lasted for approximately 1 month. In case 2, after 4 weeks of treatment, the patient experienced a considerable decrement in pain intensity with complete response after 4 months. In case 3, pain relief was achieved after 2 days, motor symptoms recovery after 2 weeks, and neuropathic features resolved completely after 5 weeks although the obturator sign resolved within 2 months.

**Lessons:**

The RALP-associated neurologic injuries may occur even when performed by highly experienced surgeons. A better understanding of the potential iatrogenic nerve lesions can surely allow an improvement in the surgical technique. A multidisciplinary approach and early multimodal pain strategy are mandatory for managing these complications.

## 1 Introduction

The robot-assisted laparoscopic prostatectomy (RALP) represents the most frequent strategy adopted for the surgical treatment of patients with localized prostate cancer. Compared to traditional retropubic radical prostatectomy, the RALP approach offers numerous and important advantages such as reduced blood loss, less postoperative pain, quicker recovery and reduced length of stay, and improvement of outcomes related to postoperative urinary continence and erectile function.^[1]^ The RALP path is associated with an overall low incidence of complications^[2]^ which ranges from 2.5% to 26%,^[3]^ depending mostly by the surgeon's experience.^[4,5]^ The most expected complications of the procedure include anastomotic leakage, bladder neck contracture, and perioperative blood loss.^[6]^ Neurologic complications can be produced by patient malpositioning on surgical table or for endopelvic damage during surgery. Nowadays, indeed, there is an awareness about potential patient positioning nerve injuries due to robotic-assisted laparoscopic surgery^[7]^ and their prevention.^[8]^ Several investigations addressed the numerical terms of this topic. For example, a retrospective analysis of 377 RALP procedures demonstrated that postoperative neuropathies occurred in 1.3%.^[9]^ More recently, Wen et al^[10]^ reported a 0.16% incidence of peripheral nerve injuries, whereas Gezginci et al^[11]^ observed neuromuscular complications in approximately 5% of patients. On the contrary, the importance of iatrogenic neurologic RALP-associated complications is often an underestimated issue in terms of rate and clinical impact. This gap is of paramount importance as these complications can often worsen the health-related quality of life (HRQoL) and can result very difficult to treat. Moreover, a better level of knowledge about the possible nerve damages intraoperatively can surely allow an improvement in the surgical technique. This report addresses 3 cases of surgical nerve lesions involving branches of the pudendal nerve (cases 1 and 2), and the left obturator nerve (case 3) due to RALP surgery. Because potential RALP-associated neurologic injuries may occur even when surgery is performed by a highly experienced surgeon, the aim is of characterizing clinical features and mechanisms of the iatrogenic damages offering suggestions for improving the surgical approach.

## 2 Case presentation

### 2.1 Premises

**Figure 1:**
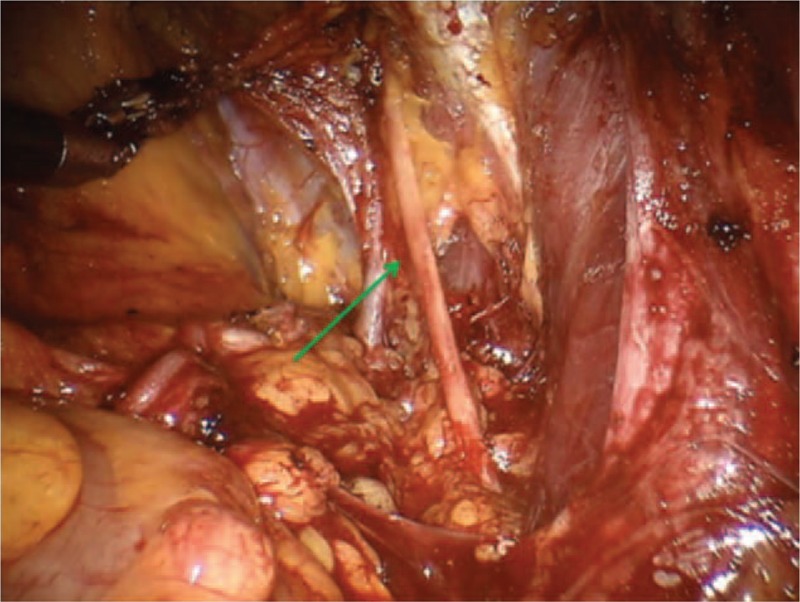
Complete exposure of the obturator nerve (green arrow).

From 2012 to March 2019, approximately 600 RALP procedures have been performed at the Istituto Nazionale Tumori, Fondazione Pascale, Naples (Italy) by a single surgeon. All cases were executed with the 4-arm da Vinci Surgical System (Intuitive Surgical Inc, Sunnyvale, CA). The standardized surgical approach follows the transperitoneal access. The 1st operative execution concerns a direct visualization of the peritoneum above the bladder. The vas deferens are then bilaterally divided under bipolar control of both deferential arteries. Blunt dissection of the fibrovascular tissue above the surface of the seminal vesicles displays the posteromedial surface of the seminal vesicle. Subsequently, the lateral side of the specimen is prepared through blunt dissection. Deep posterior dissection proceeds toward the level of the Denonvillier fascia. A key step of the procedure is the dissection at the level of both vas deferens. This approach allows a careful entry into the retroprostatic dissection in the course of the posterior bladder neck dissection. During the intervention, the umbilical ligaments and urachus are separated by using the bipolar graspers. Again, the lateral pedicles are controlled through Hem-o-lock clips (Weck surgical instruments; Teleflex Medical, Durham, NC), or titanium ligation clips (Ti-P Ligation clip Small-medium; Aesculap B. Braun Company, Tuttingen, Germany), and bipolar forceps (35 W; Valleylab Force EZ-8CS, Covidien) used distantly from the neurovascular bundles. A running stitch is performed in the Santorini plexus, and Rocco stitch is used to accost the Denonviller fascia, posterior detrusor, and posterior rhabdosphincter. The vescicourethral anastomosis is achieved with 2 running 3-0 V-Loc sutures. When indicated, pelvic lymph node dissection (PLND) is usually performed by using Hem-O-Lok clips and bipolar energy, and it includes the dissection of the obturator fossa. During this step, the obturator nerve is completely exposed (Fig. 1).

### 2.2 Case 1

A 62-year-old otherwise healthy Caucasian man (80 kg, 173 cm, body mass index [BMI] 26.7) was found to have clinical stage T2c prostate cancer as transrectal ultrasound (TRUS)-guided biopsy revealed Gleason 3 + 4 = 7, and Prostate-specific antigen (PSA) was 13.5 ng/dL). The patient underwent an uncomplicated RALP with bilateral dissection of the obturator and external iliac lymph nodes. Two days after surgery, he was discharged home. One the day after discharge, the patient was referred to the pain therapist as he complained severe pain (numeral rate scale [NRS] 6–7) located in the perianal areas. The clinical evaluation showed perianal pain, which was worsened by sitting. Paresthesia and an anal crushing sensation that impaired the patient's HRQoL were also described. Patient underwent to a multimodal therapy with the calcium channel alpha-2-delta ligand pregabalin (75 mg orally twice a day for 3 days and then 75 mg twice a day for 30 days), the association of the weak opioid tramadol plus paracetamol (film-coated tablets 37.5 mg/325 mg 3 times a day for 7 days), and l-acetyl-carnitine (1000 mg twice daily orally for 30 days). Concerning the painful features of the case, clinical results were quickly satisfactory as pain relief was achieved within 4 days. Furthermore, paresthesia resolved within 15 days. However, the anal crushing sensation lasted for approximately 1 month.

### 2.3 Case 2

A right full nerve-sparing RALP was performed in a 72-year-old man with the diagnosis of clinical T2b prostate cancer (Gleason grade group 2, PSA 12.3 ng/dL) with diabetes and obesity (BMI 31) as comorbidities. A standard PLND was performed. During the procedure, the Santorini dorsal vein complex control is usually obtained by a single running suture. In this case, the plexus and the connective tissue at the pubic symphysis were particularly represented and required an additional suture. There were no further difficulties during the rest of the procedure.

After 1 week from surgery, the patient suffered from severe burning pain (NRS at rest 7) in the perineal regions managed by nonpain medicine specialists through the nonsteroidal anti-inflammatory drug (NSAID) diclofenac (25 mg orally 4 times a day). Due to treatment failure and severity of symptoms, the case was discussed in a multidisciplinary consultation involving surgeons, pain therapists, neurologists, physical therapists, and radiologists. The video file was reviewed, although no injury such as excision, clipping, traction, or thermal damage to the nerves was found. The clinical examination showed bilateral allodynia and hyperalgesia in the perineal region, including scrotal skin and penile glans, indicating bilateral damage of the perineal nerve, a branch of the pudendal nerve. The pain was severe especially during activity (NRS incident 8). As there was no pain at the anal region, and no functional alterations of the external anal sphincter, the other branch of the pudendal nerve (inferior rectal nerve) was not injured during surgery.

Multimodal therapy consisted of pregabalin (75 mg twice daily, escalated at 150 mg twice daily after 1 week), strong opioid (tapentadol 50 mg twice daily), l-acetyl-carnitine (1000 mg twice daily orally for 30 days) and paracetamol (1000 mg orally every 8 hours). After 4 weeks of treatment, the patient reported a considerable decrement in pain intensity. Nevertheless, the functional limitation due to incident pain required a tailored physical therapy approach. Clinical evaluation showed an almost complete restoration after 4 months.

### 2.4 Case 3

A 57-year-old Caucasian man (75 kg, 173 cm, BMI 25) was found to have clinical stage T2c prostate cancer (Gleason 3 + 4 = 7 on TRUS-guided biopsy, and PSA of 18 ng/dL). The intervention of RALP and LNPD lasted approximately 2 hours and it was completed without any complication. There were no reported patient sliding issues and positioning was Trendelenburg and supine, without lithotomy position or stirrups.

After recovery from anesthesia, the patient suffered from acute pain (NRS, 7) in his left leg associated with weakness and inability to lift and adduct the limb. Clinical evaluation showed paresthesias and hypoesthesia in the sensory distribution of the obturator nerve, in the medial thigh up to the knee. The patient was treated with intravenous morphine 5 mg followed by pregabalin (50 mg twice daily), paracetamol 1000 mg, and tramadol 50 mg orally every 8 hours. Prednisone (10 mg orally for 3 days) was prescribed. This multimodal approach allowed achieving pain relief within 2 days and rapid motor symptoms recovery (2 weeks). Although neuropathic features resolved completely after 5 weeks, the obturator sign (pain induction by internal rotation of the hip against resistance)^[12]^ continued to be positive for 8 weeks and required physical therapy.

## 3 Discussion

During RALP surgery, a wide range of surgical injury to the pelvic nerves may occur. The pathophysiology of these damages recognizes different mechanisms such as compression (e.g., due to hematoma or pelvic lymphoceles), transection, incision, traction, thermal injuries, entrapment with clips. These mechanisms can be combined, and occur mostly during Santorini plexus stitch, Rocco stitch, or PLND.^[13]^ Most of these mechanisms may induce severe Schwann cell injury and demyelination, producing persistent nerve lesion.^[14]^

**Figure 2:**
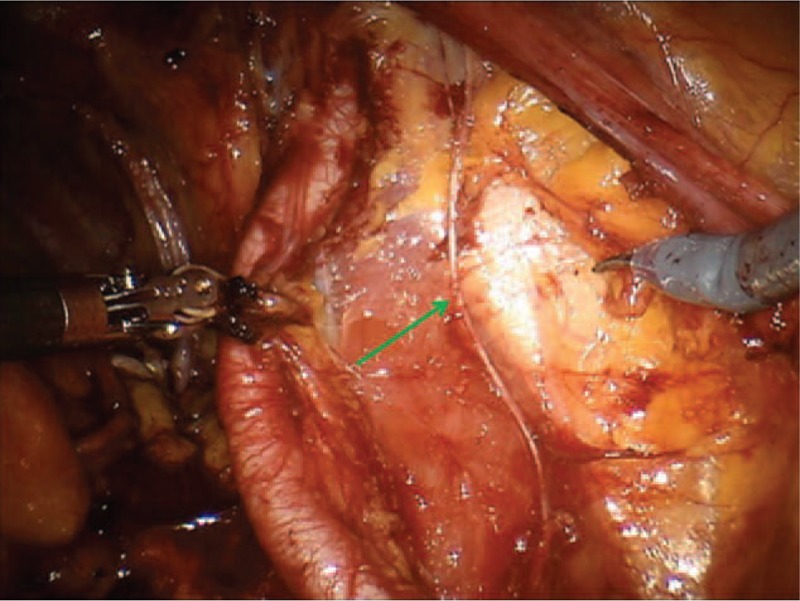
The green arrow indicates the ileo inguinal nerve.

**Figure 3:**
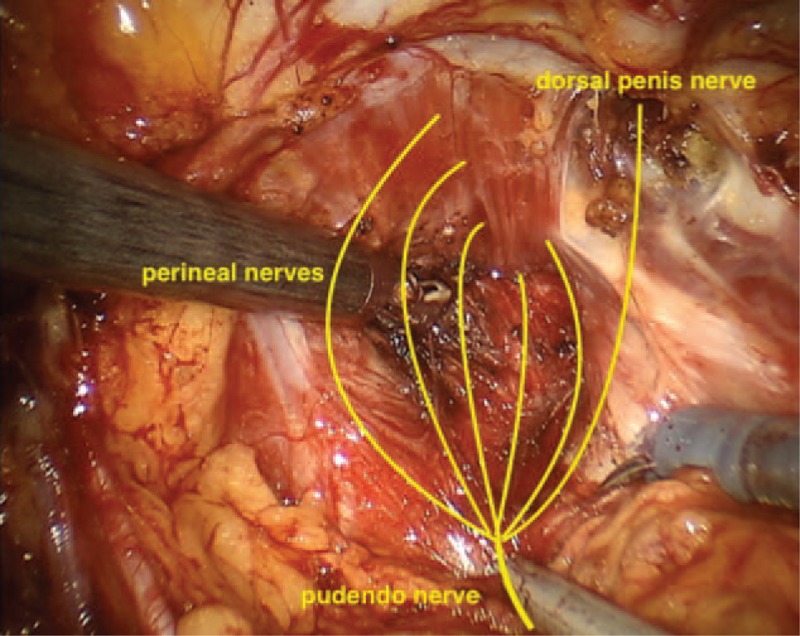
Schematic representation of the pudendal nerve at the prostatic apex.

Different nerve branches including the obturator nerve (Fig. 1), the ileoinguinal nerve (Fig. 2), and the pudendal nerve (Fig. 3) present anatomical contiguity with the prostate. The first 2 cases described concern potential injury involving different branches of the pudendal nerve. This mixed nerve arises from the ventral rami of the 2nd, 3rd, and 4th (rarely the 5th) sacral nerve roots. From its origin, the nerve proceeds downwards, passing between the piriformis and ischiococcygeal muscles, and leaves the pelvis through the greater sciatic foramen. Subsequently, it re-enters into the pelvis (lesser sciatic foramen), assuming an antero-superior orientation, and takes the pudendal canal (Alcock canal). Within the pudendal canal, it branches, giving rise to the inferior rectal nerve, which innervates the anal region and external anal sphincter, the perineal nerve, and dorsal nerve of the penis. The perineal nerve innervates the transverse perineal musculature as well as the ischiocavernosus and bulbocavernosus muscles. Furthermore, sensory afferents convey information from the scrotal skin and perineum. The dorsal nerve of the penis runs along with the lateral side of the ischio-rectal fossa, reaches the pubic symphysis and, finally, continues up to the penis (or the clitoris, in the woman).

**Figure 4:**
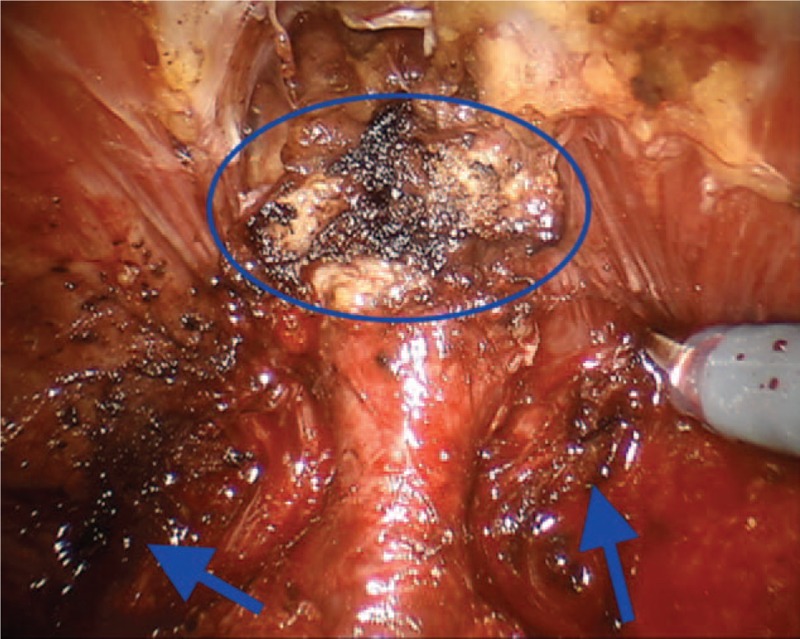
Potential damage of pudendal nerve at the periprostatic apex during thermal dissection (blue circle and arrows).

The evaluation of possible risk factors is of fundamental importance both for the prevention of complications and for their rapid identification and treatment. In case 1, the damage to the inferior rectal nerve occurred for thermal injury (Fig. 4) or traction on straight during the preparation procedure of the backplane, or during Rocco stitch. The complication was probably also related to the extent of prostatic pathology, as reported by the definitive histologic examination. The extent of the pathology, therefore, was certainly a major risk factor. Concerning the mechanism of neuropathy responsible for the clinical picture described in the second case, it can be explained as a possible injury occurred during the running stitch and the additional suture of Santorini plexus. The bilateral nerve damage may explain the severity of the neuropathic painful condition, whereas the presence of a particularly represented plexus and a connective tissue at the pubic symphysis represented a considerable risk factor. This observation must be taken into account when performing the suture.

**Figure 5:**
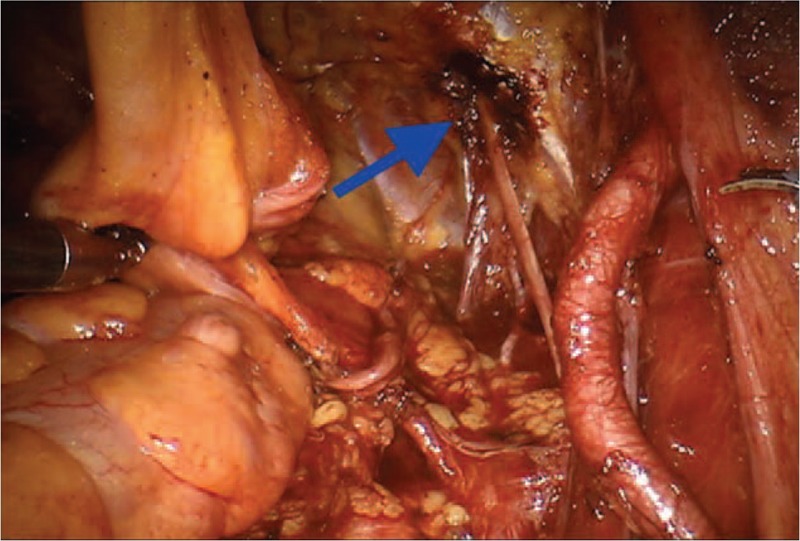
Thermal damage of the obturator nerve (blue arrow).

Case 3 regards a lesion of the left obturator nerve. This nerve originates from the ventral rami of the 2nd, 3rd, and 4th lumbar nerve roots. It follows the iliopectineal line into the lesser pelvis, runs along the lateral pelvic wall and then enters into the obturator foramen via the obturator canal. Within the canal, the nerve divides into an anterior branch, posterior branch, and a branch to the external obturator muscle then. Then, it exits through the obturator tunnel and enters the thigh. The sensory distribution of the nerve encompasses the anteromedial hip joint, the medial knee joint, and the skin on the inner thigh just above the medial knee from the anterior branch. The obturator nerve injury is described as a rare complication of robotic-assisted PLND.^[15]^ In a large series of RALPs (n. 1027), Gözen et al^[16]^ reported 2 cases (0.2%) of nerve transections. Because the obturator nerve can be adherent to lymph nodes or enclosed by them, a careful nerve mobilization should be performed, and fixed lymph nodes should not be mobilized roughly. Specifically, the proximal part of the obturator nerve runs closely the external iliac vein and the internal iliac artery. This is the location of the internal iliac lymph nodes. In our case, we suppose that the nerve damage was caused during the extensive PLND through a thermal injury of the nerve at the entrance of the obturator fossa (Fig. 5). Alternatively, a compressive effect on the nerve was produced by a PLND-induced lymphocele. The combination of both mechanisms seems to be another plausible explanation. According to this latter hypothesis, the early multimodal pain strategy and antiedematous therapy may explain the rapid resolution of the clinical picture. Furthermore, since in this case, it is possible to identify risk factors related to the procedure, the knowledge of pathogenetic mechanisms certainly has considerable clinical value for the prevention of complications.

**Figure 6:**
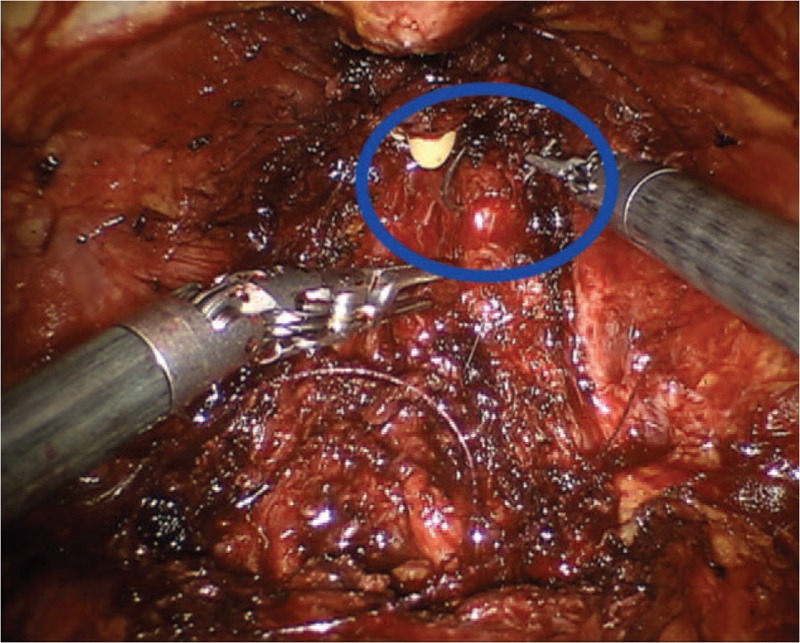
The Rocco stitch used to accost the Denonviller fascia, posterior detrusor, and posterior rhabdosphincter is performed not at full thickness.

Several suggestions focused on improving surgical technique and aimed at avoiding neurologic complications can be proposed. Firstly, the thermal energy should be minimized by using bipolar output energy <35 and <50 W in monopolar. Furthermore, hemostasis through microsutures (e.g., 4-0 brainded absorbable suture CV-25 TAPER 1/2 circle 17 mm Polysorb; Covidien) can represent a less invasive approach. Other suggestions concern the use of titanium clip during dissection, Rocco stitch (e.g., 3-0 V-Loc barbed absorbable suture GU-46 TAPER 5/8 circle 27 mm; Covidien) performed not through full-thickness modality (Fig. 6). Finally, because the different branches of the pudendal nerve run laterally and dorsal to the rectum it should be recommended to minimize traction maneuvers during the procedure of prostate detachment.

About the clinical impact of RALP-induced nerve damages, the Clavien classification system, a widely used tool to classify surgical complications, is commonly used to evaluate the severity of RALP complications.^[17]^ According to this approach, the neurologic complications reported in this series should be defined as grade I complications (Any deviation from the normal postoperative course, bedside wound debridement, basic pharmacologic therapy, or expectant management required). Nevertheless, in 1 case, there was impairment in patient's HRQoL (case 1); in the 2nd patient, the complication brought about a great deal of distress in the patient and its management required high level of care through the involvement of a multidisciplinary team composed of pain therapists, physical therapists, neurologists and nurses, and, in turn, high health care costs. Furthermore, case 3 was characterized by severe acute pain. As a consequence, it should be provided an update of the Clavien classification by introducing criterion(s) focused on disability, the grade of painful experience, or HRQoL impairment. Again, because in all 3 cases the involvement of pain therapists and other care professionals was a winning strategy, an early patient-tailored multimodal therapy is mandatory for managing these complications.^[18]^

## 4 Conclusion

The RALP-associated neurologic injuries may occur even when performed by highly experienced surgeons. Because of the lack of data on the topic represents a serious issue, this report can allow stimulating further studies for obtaining data on potential risk factors (e.g., BMI, previous operations, intraoperative, and anatomical conditions), for dissecting clinical features and mechanisms of possible nerve injuries during RALP, and, in turn, for optimizing surgical approach and outcomes.

## Acknowledgment

The authors thank Dr Luigi Claudio for image acquisition.

## Author contributions

**Conceptualization:** Marco Cascella, Arturo Cuomo, Sisto Perdonà.

**Data curation:** Marco Cascella, Alessandro Izzo, Luigi Castaldo.

**Investigation:** Marco Cascella.

**Methodology:** Sisto Perdonà.

**Supervision:** Marco Cascella, Arturo Cuomo.

**Validation:** Giovanni Grimaldi, Barbara Di Caprio, Paola Del Prete.

**Writing – original draft:** Giuseppe Quarto, Sabrina Bimonte.

**Writing – review & editing:** Marco Cascella, Raffaele Muscariello.

marco cascella orcid: 0000-0002-5236-3132.
